# Comparison of drug-eluting bead with conventional transcatheter arterial chemoembolization for hepatocellular carcinoma with portal vein tumor thrombus: a randomized clinical trial

**DOI:** 10.1097/JS9.0000000000001691

**Published:** 2024-05-22

**Authors:** Tan-Yang Zhou, Guo-Fang Tao, Guan-Hui Zhou, Yue-Lin Zhang, Tong-Yin Zhu, Sheng-Qun Chen, Hong-Liang Wang, Bao-Quan Wang, Li Jing, Feng Chen

**Affiliations:** a Hepatobiliary and Pancreatic Interventional Treatment Center, Division of Hepatobiliary and Pancreatic Surgery; b Department of Nursing, The First Affiliated Hospital, Zhejiang University School of Medicine; c Department of Radiology, The First Affiliated Hospital, Zhejiang University School of Medicine; d Zhejiang Provincial Research Center for Diagnosis and Treatment of Hepatobiliary Diseases, Hangzhou, Zhejiang Province, China

**Keywords:** drug-eluting bead, hepatocellular carcinoma, transarterial chemoembolization

## Abstract

**Background:**

Drug-eluting bead transarterial chemoembolization (DEB-TACE) has shown efficacy for treating hepatocellular carcinoma (HCC) with portal vein tumor thrombus (PVTT). However, whether DEB-TACE is superior to conventional TACE (cTACE) remains unclear.

**Objective:**

This randomized controlled trial aimed to compare the efficacy and safety of DEB-TACE versus cTACE in treating HCC with PVTT.

**Methods:**

The study was conducted at a tertiary care center in Southeast China. HCC patients with PVTT were randomized at a 1:1 ratio into the DEB-TACE or cTACE groups. The primary endpoint was progression-free survival (PFS), and the secondary endpoints were overall survival (OS) and the incidence of adverse events (AEs). An independent review committee assessed the radiologic response according to the modified Response Evaluation Criteria in Solid Tumors (mRECIST). AEs were assessed by the Common Terminology Criteria for Adverse Events (CTCAE) version 4.0. Systemic therapies were not restricted.

**Results:**

Between September 2018 and July 2020, 163 patients were randomized to undergo DEB-TACE (*n*=82) or cTACE (*n*=81). Nine patients were excluded, and 154 patients were included in the final analysis; the median age was 55 years (range, 24–78 years), and 140 (90.9%) were male. The median PFS in the DEB-TACE group was 6.0 months (95% CI, 5.0–10.0) versus 4.0 months (95% CI, 3.0–5.0) in the cTACE group (hazard ratio, 0.63; 95% CI, 0.42–0.95; *P*=0.027). The DEB-TACE group showed a higher response rate [51 (66.2%) vs. 36 (46.8%); *P*=0.0015] and a longer median OS [12.0 months (95% CI, 9.0–16.0) vs. 8.0 months (95% CI, 7.0–11.0), *P*=0.039] than the cTACE group. Multivariate analysis showed that the treatment group, ALBI score, distant metastasis and additional TKIs were the four independent prognostic factors correlated with PFS. In addition, the treatment group, PVTT group and combination with surgery were independently associated with OS. AEs were similar in the two groups, and postembolization syndrome was the most frequent AE.

**Conclusion:**

DEB-TACE is superior to cTACE in treating HCC patients with PVTT, demonstrating improved PFS and OS with an acceptable safety profile, and may thus emerge as a promising treatment strategy for HCC patients with PVTT.

**Trial registration:**

Chinese Clinical Trial Registry ChiCTR1800018035.

## Graphical abstract

**Figure:**
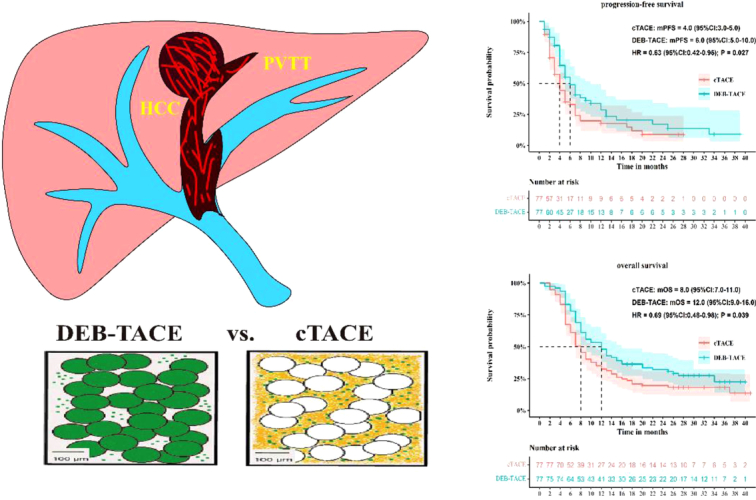

## Introduction

Highlights

- Transarterial chemoembolization (TACE) is a relatively safe and effective therapeutic approach for hepatocellular carcinoma (HCC) patients with portal vein tumor thrombu (PVTT).
- Drug-eluting bead (DEB)-TACE is superior to conventional TACE (cTACE) in treating HCC patients with PVTT.
- The treatment group and ALBI grade are closely related to progression-free survival (PFS), while the treatment group and PVTT classification are key factors determining overall survival (OS).

Portal vein tumor thrombosis (PVTT) is a prevalent occurrence in hepatocellular carcinoma (HCC) and serves as a crucial indicator of a poor prognosis^1,2^. Despite advances in the treatment of HCC, managing HCC with PVTT remains challenging^3,4^. According to Western guidelines^5,6^, HCC patients with PVTT are considered to have minimal chances for a cure, and the only anticancer treatment option is systemic therapy, which results in a median overall survival of 6.5months^7^. However, treatment strategies for these patients differ from Western approaches in the Asia-Pacific region^8,9^ where more aggressive anticancer treatments are recommended, and promising survival outcomes have been reported with surgical resection, radiotherapy, transarterial chemoembolization (TACE), and other modalities^10^.

TACE is an effective locoregional treatment for HCC with PVTT^11^ and the latest Chinese guidelines have proposed TACE as an alternative therapy for these patients^8,12^. Nevertheless, conventional lipiodol-based TACE (cTACE) has low treatment response rates, with a median overall survival (OS) of only 4.0–6.1 months^13^. Drug-eluting bead (DEB) has emerged as innovative drug-delivering agents for TACE, enabling higher local drug concentrations within the targeted tumor and lower systemic concentrations compared to cTACE. However, the clinical superiority of DEB-TACE in terms of treatment response and survival benefits remains a subject of debate. Our previous work has demonstrated that DEB-TACE using CalliSpheres was efficient and well-tolerated in HCC patients^14^ and safe in HCC patients with PVTT, yielding favorable preliminary clinical outcomes^15^. Nonetheless, no large randomized controlled trials have been conducted to compare the efficacy and safety of DEB-TACE versus cTACE in the treatment of HCC with PVTT in clinical practice. Thus, we initiated this trial at our center.

## Methods

### Study design and patients

This prospective, randomized, controlled, unblinded trial was conducted at a tertiary care hospital to evaluate the efficacy and safety of DEB-TACE in treating HCC patients with PVTT. The primary endpoint was progression-free survival (PFS), assessed via modified Response Evaluation Criteria in Solid Tumors (mRECIST) by an independent review committee (IRC). The secondary endpoints were OS and the incidence of adverse events (AEs). The study was approved by the Institutional Review Board (IRB No. 2018-804), and all study participants provided written informed consent. The study protocol was registered at chictr.org.cn prior to the start of participant enrollment. Written informed consent was obtained from the patient for publication of this study. A copy of the written consent is available for review by the Editor-in-Chief of this journal upon request. The study has been reported in accordance with Consolidated Standards of Reporting Trials (CONSORT) Guidelines^16^.

Inclusion criteria required patients to be aged 18–80 years; have a diagnosis of HCC with PVTT without previous treatment; ineligible for surgical resection; have Child-Pugh A or B7 liver disease; have an ECOG Performance Status of 0–1; and have adequate hematological, liver, and renal functions. Adequate functions were defined as follows: hemoglobin level greater than or equal to 90 g/L; absolute neutrophil count greater than or equal to 1.5×10^9^/L; platelet count greater than or equal to 50×109/l; alanine aminotransferase (ALT) and aspartate aminotransferase (AST) levels 5-fold or less of the upper limit of normal (ULN); serum total bilirubin level less than or equal to 2-fold ULN; serum creatinine level less than or equal to 1.5-fold ULN; and serum albumin level greater than or equal to 30 g/l. The key exclusion criteria included known fibrolamellar HCC, sarcomatoid HCC, cholangiocarcinoma or mixed cholangiocarcinoma and HCC; massive hepatic arteriovenous fistula; a history of previous esophageal variceal bleeding; coexistent other malignant tumors; and uncontrolled infection or HIV.

HCC was diagnosed based on histological or image-derived EASL criteria^6^. PVTT was determined by three radiologists with over five years of experience in imaging diagnosis. On computed tomography (CT) or MRI, the diagnosis of PVTT was made based on the presence of a low-attenuation mass within the portal vein and the mass arterial phase enhancement^17^. The extent of PVTT was classified according to Cheng’s classification^8^.

### Procedures

The patients were randomly assigned in a 1:1 ratio to either the DEB-TACE or cTACE treatment group. We created the computer-generated random number using a Microsoft Excel sheet and coded control as ‘cTACE’ and study as ‘DEB-TACE’. Afterward, we prepared envelopes according to a random number and allocated participants to either control or study group based on the random number. We used an envelope to minimize researcher selection bias. Random numbers were kept in an envelope. Upon confirming a participant’s eligibility, the next envelope in the sequence was opened, and the intervention or control allocation was entered on a randomization list. Stratification of the randomization was performed according to the grade of portal vein invasion (type Ⅰ/Ⅱ versus type Ⅲ/Ⅵ).

Under local anesthesia, TACE was performed according to a standard protocol, via femoral artery access with a 5-F catheter and selective catheterization of the tumors’ feeding arteries with a 2.4-F or 2.7-F microcatheter, depending upon the liver involvement and the vascular anatomy. Cone-beam computed tomography (CBCT) was routinely used to visualize the tumor-feeding vessels and for immediate post-embolization assessment. For cTACE, 60 mg of doxorubicin was administered as an emulsion with 10–20 ml iodized oil (Lipiodol; Guerbet, Villepinte, France) and slowly injected into the tumor-feeding artery under fluoroscopic guidance, followed by embolization with absorbable gelatin sponge particles (Alicon). For DEB-TACE, CalliSphere beads (Jiangsu Hengrui Pharmaceutical Co., Ltd) sized 100–300 μm were loaded with 60 mg of doxorubicin per vial and administered intra-arterially after mixing with nonionic contrast medium, up to a maximum of one vial; additional embolization was performed with non-resorbable bland microparticles when needed. Substantial arterial flow reduction to the tumor was defined as the technical endpoint of embolization, measured by the time it took for the contrast column to clear (typically 2–5 heartbeats)^18^.

Follow-up appointments were scheduled one week after TACE, during which clinical assessments and laboratory tests were performed. Patients underwent a multiphasic contrast-enhanced MRI/CT scan a month post-TACE to assess the response. The mRECIST was used to assess the therapeutic effects on the primary liver tumor^19^. The assessment of PVTT response was conducted using the PVTT classification system, with adjustments made based on mRECIST criteria. Specifically, any downstaging in Cheng’s PVTT classification accompanied by partial recanalization of the portal vein was considered partial remission (PR), whereas any upstaging in the PVTT classification was considered progressive disease (PD). The assessment of overall therapeutic response was determined as PD if either the primary tumor or PVTT was classified as PD. Conversely, it was defined as PR if either the primary tumor or PVTT was classified as PR and the other did not progress. If both the primary tumor and PVTT were classified as complete response (CR) or stable disease (SD), it was defined as CR or SD, respectively. The overall objective response rate (ORR) was defined as the proportion of confirmed CR or PR at the best response. DCR was defined as the percentage of confirmed CR, PR or SD at the best response. If the response was inadequate, additional TACE procedures were planned. Alternatively, if the response was adequate, patients were monitored for disease progression with 3-month imaging studies. Patients were allowed to use systemic therapies and traditional Chinese medicine (TCM) treatment during the study. Systemic therapies such as tyrosine kinase inhibitors (TKIs)or/and immune checkpoint inhibitors (ICIs) were prescribed for patients with distant metastasis within one week after initial TACE provided that liver function had been restored. After downstaging, patients who met the resectable criteria^20^ were considered for hepatectomy or salvage liver transplantation. Patients were followed up for 2 years. The time between the initial TACE treatment and disease progression or death was used to evaluate PFS or OS. The ratio of tumor volume to total liver volume, as assessed by CT/MRI, was used to calculate the liver tumor burden (LTB).

AEs were closely monitored and recorded throughout the trial, with assessments conducted during and after each treatment, as well as at all follow-up visits. Any arising AEs were graded according to the NCI Common Terminology Criteria for Adverse Events (CTCAE) version 4.0. AEs occurring within two weeks of TACE were deemed treatment-related, whereas AEs after this period were reported only if a causal correlation was suspected.

### Statistical analysis

The sample size was estimated based on the assumption of a median PFS of 5 months and 3 months for patients receiving DEB-TACE or cTACE treatment, respectively^15,21,22^. To achieve 80% power and a two-sided α of 0.05, it calculated that 128 patients needed to be enrolled, with a 24-month enrollment and follow-up period. Accounting for an estimated dropout rate of 5%, our target enrollment was set at 163 patients (81 in the cTACE group and 82 in the DEB-TACE group).

The primary efficacy analysis was conducted in both the intent-to-treat (ITT) and per-protocol (PP) populations, while the safety analysis included all randomized patients who received at least one session of protocol treatment. Results were presented as mean (SD), number (%), or median (95% CI) and were compared using Student’s *t*-tests or χ^2^ tests. Survival outcomes were calculated using the Kaplan–Meier method and compared using the log-rank test. Hazard ratios (HRs) and 95% CIs highlighted differences between the two groups. All *P* values were two-sided, and those below 0.05 were considered statistically significant. Statistical analyses were performed using IBM SPSS Statistics (version 25.0, IBM) and supplemented with the R software package (version 4.2.2, R Foundation for Statistical Computing).

## Results

### Patient characteristics and treatment

**Figure 1:**
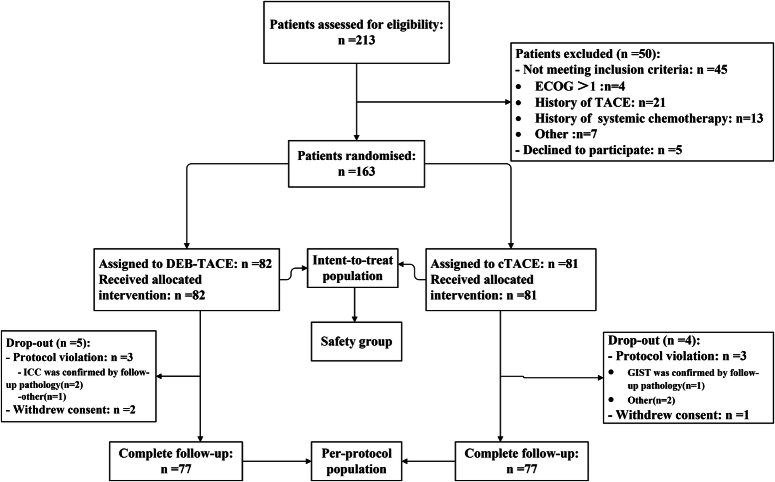
Summary flow chart of the study. cTACE, conventional transarterial chemoembolization; DEB-TACE, drug-eluting bead TACE; ECOG, Eastern Cooperative Oncology Group;

Between September 2018 and July 2020, 213 patients diagnosed with HCC underwent screening, of which 163 were ultimately enrolled in the study. A flow chart describing in detail the patient selection process is illustrated in Figure 1. The follow-up period was extended until July 28, 2022. In the ITT population, 82 patients [median age, 55.0 years; interquartile range (IQR), 49.0–63.8 years; 74 men (90.2%)] were randomized to receive DEB-TACE, while 81 [median age, 55.0 years; IQR, 50.0–62.0 years; 75 men (92.6%)] were assigned to cTACE (Table 1). All patients received their assigned treatment. However, five patients in the DEB-TACE group and four in the cTACE group were deemed ineligible and excluded from further analysis (Fig. 1). The PP population subsequently consisted of 77 patients in the DEB-TACE group [median age, 55.0 years; IQR, 49.0–63.0 years; 69 men (89.6%)] and 77 in the cTACE group [median age, 55.0 years; IQR, 50.0–62.0 years; 71 men (92.2%)]. Baseline characteristics between the randomized groups were well-matched (Table 2). Patients in the two groups equally received a median of 2.0 sessions (range, 1–7 sessions) of TACE. Hepatectomy or salvage liver transplantation was performed in 18 (23.4%) patients in the DEB-TACE group and 11 (14.3%) patients in the cTACE group after downstaging. Detailed information on the treatments is summarized in Table 3.

**Table 1:** Demographic and clinical characteristics for ITT population.

	ALL (*N*=163)	cTACE (*N*=81)	DEB-TACE (*N*=82)	*P*
Age (year): median (range)	55.0 [49.5; 63.0]	55.0 [50.0; 62.0]	55.0 [49.0; 63.8]	0.852
Sex, *N* (%)				0.798
Female	14 (8.59)	6 (7.41)	8 (9.76)	
Male	149 (91.4)	75 (92.6)	74 (90.2)	
ECOG performance status, *N* (%)				0.568
0	67 (41.1)	31 (38.3)	36 (43.9)	
1	96 (58.9)	50 (61.7)	46 (56.1)	
Child-Pugh stage, *N* (%)				0.499
A	137 (84.0)	66 (81.5)	71 (86.6)	
B	26 (16.0)	15 (18.5)	11 (13.4)	
HBV infection, *N* (%)				0.239
No	22 (13.5)	14 (17.3)	8 (9.76)	
Yes	141 (86.5)	67 (82.7)	74 (90.2)	
Tumor number, *N* (%)				0.379
Single	71 (43.6)	32 (39.5)	39 (47.6)	
Multiple	92 (56.4)	49 (60.5)	43 (52.4)	
ALBI score, *N* (%)				0.333
Grade 1	73 (47.4)	33 (42.9)	40 (51.9)	
Grade 2	81 (52.6)	44 (57.1)	37 (48.1)	
Maximum tumor size (cm)	9.37±3.82	9.92±3.82	8.83±3.77	0.069
Tumor size group, *N* (%)				0.214
≤5 cm	18 (11.0)	7 (8.64)	11 (13.4)	
≤10 cm	83 (50.9)	38 (46.9)	45 (54.9)	
>10 cm	62 (38.0)	36 (44.4)	26 (31.7)	
LTB, *N* (%)				0.098
≤50%	94 (57.7)	41 (50.6)	53 (64.6)	
>50%	69 (42.3)	40 (49.4)	29 (35.4)	
PVTT type, *N* (%)				0.226
I	54 (33.1)	25 (30.9)	29 (35.4)	
II	53 (32.5)	29 (35.8)	24 (29.3)	
III	49 (30.1)	26 (32.1)	23 (28.0)	
IV	7 (4.29)	1 (1.23)	6 (7.32)	
PVTT group, *N* (%)				0.914
I/II	107 (65.6)	54 (66.7)	53 (64.6)	
III/IV	56 (34.4)	27 (33.3)	29 (35.4)	
PVTT supply, *N* (%)				0.691
Poor	87 (53.4)	45 (55.6)	42 (51.2)	
Rich	76 (46.6)	36 (44.4)	40 (48.8)	
Hepatic vein invasion, *N* (%)				0.096
No	72 (44.2)	30 (37.0)	42 (51.2)	
Yes	91 (55.8)	51 (63.0)	40 (48.8)	
Distant metastasis, *N* (%)				0.577
No	111 (68.1)	53 (65.4)	58 (70.7)	
Yes	52 (31.9)	28 (34.6)	24 (29.3)	
AFP group, *N* (%)				0.588
≤20 ng/ml	38 (23.3)	17 (21.0)	21 (25.6)	
<400 ng/ml	41 (25.2)	19 (23.5)	22 (26.8)	
≥400 ng/ml	84 (51.5)	45 (55.6)	39 (47.6)	
WBC (10^9^/l): median (range)	5.70 [4.65; 7.20]	5.80 [4.60; 7.40]	5.70 [4.70; 6.77]	0.414
RBC (10^12^/l): median (range)	4.36 [3.89; 4.82]	4.38 [3.89; 4.81]	4.35 [3.79; 4.83]	0.977
HGb (g/l): median (range)	135 [120; 148]	134 [121; 148]	136 [118; 149]	0.679
PLT (10^9^/l): median (range)	171 [121; 224]	174 [127; 225]	162 [118; 220]	0.369
ALB (g/l): median (range)	39.4 [35.7; 43.2]	39.0 [35.2; 42.4]	39.7 [35.9; 44.3]	0.153
TBIL(μmol/l): median (range)	16.0 [12.4; 24.0]	17.0 [11.4; 24.8]	15.8 [13.6; 23.5]	0.900
ALT (U/l): median (range)	41.0 [26.5; 53.5]	41.0 [27.0; 48.0]	42.0 [25.2; 57.0]	0.793
AST (U/l): median (range)	54.0 [39.0; 81.0]	58.0 [42.0; 81.0]	53.0 [37.0; 80.2]	0.369
Cr (μmol/l): median (range)	71.0 [64.0; 83.0]	72.0 [64.0; 82.0]	70.0 [63.2; 83.0]	0.964
PT (s): median (range)	12.7 [12.1; 13.3]	12.8 [12.0; 13.4]	12.6 [12.1; 13.2]	0.632

Data reported as No. (%) unless otherwise indicated.

AFP, alpha-fetoprotein; ALB, albumin; ALBI grade, Albumin-Bilirubin grade; ALT, alanine aminotransferase; AST, aspartate aminotransferase; Cr, creatine; cTACE, conventional transarterial chemoembolization; DEB-TACE, drug-eluting bead TACE; ECOG, Eastern Cooperative Oncology Group; ITT, intent-to-treat; LTB, liver tumor burden; PLT, platelet count; PT, prothrombin time; PVTT, portal vein tumor thrombosis; RBC, red blood cell; TBIL, total bilirubin; WBC, white blood cell.

**Table 2:** Demographic and clinical characteristics for PP population.

Characteristic	ALL (*N*=154)	cTACE (*N*=7*7*)	DEB-TACE (*N*=77)	*P*
Age (year): median (range)	55.0 [50.0; 62.8]	55.0 [50.0; 62.0]	55.0 [49.0; 63.0]	0.685
Sex, *N* (%)				0.779
Female	14 (9.09)	6 (7.79)	8 (10.4)	
Male	140 (90.9)	71 (92.2)	69 (89.6)	
ECOG performance status, *N* (%)				0.507
0	59 (38.3)	27 (35.1)	32 (41.6)	
1	95 (61.7)	50 (64.9)	45 (58.4)	
Child-Pugh stage, *N* (%)				0.651
A	131 (85.1)	64 (83.1)	67 (87.0)	
B	23 (14.9)	13 (16.9)	10 (13.0)	
HBV infection, *N* (%)				0.250
No	22 (14.3)	14 (18.2)	8 (10.4)	
Yes	132 (85.7)	63 (81.8)	69 (89.6)	
Tumor number, *N* (%)				0.256
Single	68 (44.2)	30 (39.0)	38 (49.4)	
Multiple	86 (55.8)	47 (61.0)	39 (50.6)	
ALBI score, *N* (%)				0.333
Grade 1	73 (47.4)	33 (42.9)	40 (51.9)	
Grade 2	81 (52.6)	44 (57.1)	37 (48.1)	
Maximum tumor size (cm)	9.41±3.79	9.83±3.78	9.00±3.79	0.177
Tumor size group, *N* (%)				0.411
≤5 cm	16 (10.4)	7 (9.09)	9 (11.7)	
≤10 cm	78 (50.6)	36 (46.8)	42 (54.5)	
>10 cm	60 (39.0)	34 (44.2)	26 (33.8)	
LTB, *N* (%)				0.101
≤50%	91 (59.1)	40 (51.9)	51 (66.2)	
>50%	63 (40.9)	37 (48.1)	26 (33.8)	
PVTT type, *N* (%)				0.337
I	53 (34.4)	25 (32.5)	28 (36.4)	
II	52 (33.8)	29 (37.7)	23 (29.9)	
III	43 (27.9)	22 (28.6)	21 (27.3)	
IV	6 (3.90)	1 (1.30)	5 (6.49)	
PVTT group, *N* (%)				0.729
I/II	105 (68.2)	54 (70.1)	51 (66.2)	
III/IV	49 (31.8)	23 (29.9)	26 (33.8)	
PVTT supply, *N* (%)				0.418
Poor	84 (54.5)	45 (58.4)	39 (50.6)	
Rich	70 (45.5)	32 (41.6)	38 (49.4)	
Hepatic vein invasion, *N* (%)				0.192
No	65 (42.2)	28 (36.4)	37 (48.1)	
Yes	89 (57.8)	49 (63.6)	40 (51.9)	
Distant metastasis, *N* (%)				0.602
No	106 (68.8)	51 (66.2)	55 (71.4)	
Yes	48 (31.2)	26 (33.8)	22 (28.6)	
AFP group, *N* (%)				0.626
≤20 ng/ml	37 (24.0)	17 (22.1)	20 (26.0)	
<400 ng/ml	39 (25.3)	18 (23.4)	21 (27.3)	
≥400 ng/ml	78 (50.6)	42 (54.5)	36 (46.8)	
WBC (10^9^/l): median (range)	5.70 [4.62; 7.18]	5.80 [4.50; 7.30]	5.70 [4.70; 6.80]	0.535
RBC (10^12^/l): median (range)	4.36 [3.89; 4.82]	4.38 [3.89; 4.81]	4.35 [3.73; 4.84]	0.961
HGb (g/l): median (range)	136 [120; 149]	136 [124; 149]	137 [117; 149]	0.837
PLT (10^9^/l): median (range)	168 [121; 220]	173 [127; 223]	161 [118; 219]	0.412
ALB (g/l): median (range)	39.4 [35.8; 43.1]	39.0 [35.2; 42.4]	39.9 [36.4; 43.6]	0.126
TBIL(μmol/l): median (range)	16.2 [12.1; 24.1]	17.0 [11.1; 24.8]	15.8 [13.5; 23.5]	0.938
ALT (U/l): median (range)	42.0 [27.0; 53.8]	41.0 [27.0; 48.0]	43.0 [26.0; 57.0]	0.752
AST (U/l): median (range)	53.5 [39.2; 78.8]	58.0 [42.0; 81.0]	53.0 [37.0; 78.0]	0.357
Cr (μmol/l): median (range)	72.0 [64.0; 83.0]	72.0 [64.0; 82.0]	71.0 [64.0; 84.0]	0.876
PT (s): median (range)	12.8 [12.1; 13.3]	12.8 [12.0; 13.5]	12.6 [12.1; 13.2]	0.498

Data reported as No. (%) unless otherwise indicated.

AFP, alpha-fetoprotein; ALB, albumin; ALBI grade, Albumin-Bilirubin grade; ALT, alanine aminotransferase; AST, aspartate aminotransferase; Cr, creatine; cTACE, conventional transarterial chemoembolization; DEB-TACE, drug-eluting bead TACE; ECOG, Eastern Cooperative Oncology Group; LTB, liver tumor burden; PLT, platelet count; PP, per-protocol; PT, prothrombin time; PVTT, portal vein tumor thrombosis; RBC, red blood cell; TBIL, total bilirubin; WBC, white blood cell.

**Table 3:** Details of the treatments for patients in the two group in PP population.

Variants	ALL *N*=154, *N* (%)	cTACE, *N*=77, *N* (%)	DEB-TACE, *N*=77, *N* (%)	*P*
TKIs				1.000
No	72 (46.8)	36 (46.8)	36 (46.8)	
Yes	82 (53.2)	41 (53.2)	41 (53.2)	
ICIs				0.620
No	94 (61.0)	49 (63.6)	45 (58.4)	
Yes	60 (39.0)	28 (36.4)	32 (41.6)	
TKIs or ICIs				0.398
No	54 (35.1)	30 (39.0)	24 (31.2)	
Yes	100 (64.9)	47 (61.0)	53 (68.8)	
TKIs and ICIs				0.856
No	112 (72.7)	55 (71.4)	57 (74.0)	
Yes	42 (27.3)	22 (28.6)	20 (26.0)	
TCM				1.000
No	97 (63.0)	48 (62.3)	49 (63.6)	
Yes	57 (37.0)	29 (37.7)	28 (36.4)	
SBRT				0.080
No	129 (83.8)	69 (89.6)	60 (77.9)	
Yes	25 (16.2)	8 (10.4)	17 (22.1)	
Combined surgery				0.104
No	125 (81.2)	66 (85.7)	59 (76.6)	
Hepatectomy	21 (13.6)	6 (7.8)	15 (19.5)	
LT	8 (5.2)	5 (6.5)	3 (3.9)	
TACE sessions				0.892
1	82 (53.2)	40 (51.9)	42 (54.5)	
2	29 (18.8)	16 (20.8)	13 (16.9)	
3	25 (16.2)	13 (16.9)	12 (15.6)	
≥4	18 (11.7)	8 (10.4)	10 (13.0)	

cTACE, conventional transarterial chemoembolization; DEB-TACE, drug-eluting bead TACE; ICIs, immune checkpoint inhibitors; LT, liver transplantation; PP, per-protocol; SBRT, stereotactic body radiation therapy; TCM, Traditional Chinese Medicine; TKIs, tyrosine kinase inhibitors.

### Efficacy

**Figure 2:**
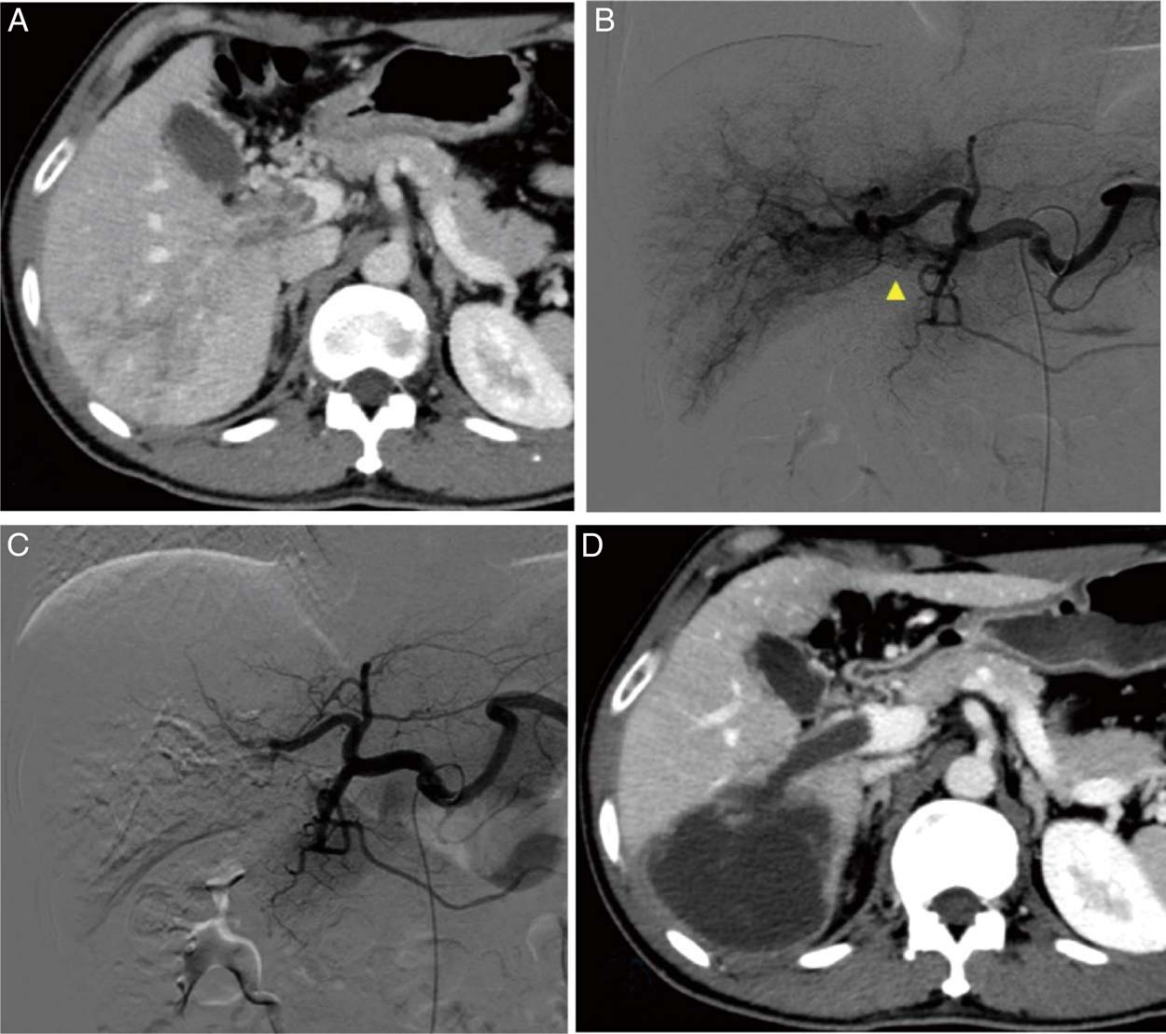
A typical case of DEB-TACE treating hepatocellular carcinoma (HCC) with PVTT. (A) Contrast-enhanced portal venous phase CT scan in the axial plane shows the infiltrative HCC in the S6 hepatic segment with PVTT in the right branch of the portal vein and the main portal vein (type Ⅲ). (B) DSA of the common hepatic artery shows a large irregular tumor stain in segment 6 and streaklike tumor blood vessels (yellow arrow) in PVTT. (C) DSA after DEB-TACE shows tumor and PVTT staining disappeared. (D) Enhanced CT images in the portal venous phase indicate complete necrosis of both the tumor and the PVTT one month after D-TACE treatment. CT, computed tomography; DEB-TACE, drug-eluting bead transarterial chemoembolization; PVTT, portal vein tumor thrombus.

According to the mRECIST, the overall ORR was significantly higher in the DEB-TACE group than in the cTACE group (63.4% vs. 44.4% in the ITT population and 66.2% vs. 46.8% in the PP population, all *P*=0.015). CR was observed in five patients in the DEB-TACE group, and two patients in the cTACE group (Fig. 2). The overall DCR was similar between the groups ( 91.5% vs. 85.2% in the ITT population and 92.2% vs. 85.7% in the PP population, *P*=0.21 and 0.20, respectively) (Table 4).

**Table 4:** The radiologic response rate of treatment groups.

	PP population			ITT population		
Characteristic	cTACE *N*=77	DEB-TACE *N*=77	*P*	cTACE *N*=81	DEB-TACE *N*=82	*P*
Best response, *N* (%)			0.087			0.089
CR	2 (2.6)	5 (6.5)		2 (2.5)	5 (6.1)	
PR	34 (44.2)	46 (59.7)		34 (42.0)	47 (57.3)	
SD	30 (39.0)	20 (26.0)		33 (40.7)	23 (28.0)	
PD	11 (14.3)	6 (7.8)		12 (14.8)	7 (8.5)	
ORR, *N* (%)	36 (46.8)	51 (66.2)	0.015	36 (44.4)	52 (63.4)	0.015
DCR, *N* (%)	66 (85.7)	71 (92.2)	0.20	69 (85.2)	75 (91.5)	0.21

cTACE, conventional transarterial chemoembolization; DEB-TACE, drug-eluting bead TACE; CR, complete response; DCR, disease control rate; ITT, intent-to-treat; ORR, objective response rate; PD, progressive disease; PP, per-protocol; PR, partial response; SD, stabile disease.

**Figure 3:**
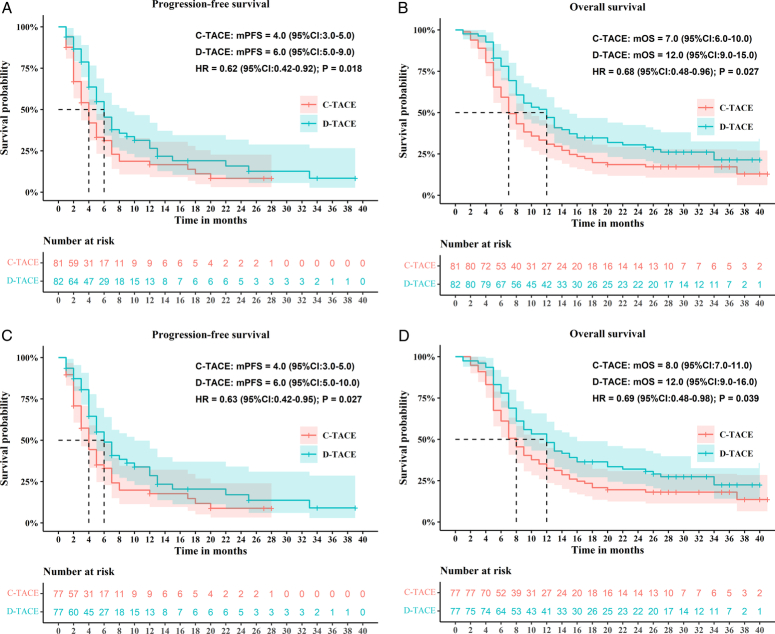
Kaplan–Meier plots of median PFS and OS in the ITT (A, B) and PP (C, D) population. Efficacy outcomes in participants in the drug-eluting bead transarterial chemoembolization group versus cTACE group for the treatment of hepatocellular carcinoma with portal vein tumor thrombus. Kaplan–Meier plots show PFS and OS in the ITT population (A, B) and the PP population (C, D). Note: *P* values were calculated by using the log-rank test. Dashed lines indicate 95% CIs. cTACE, conventional transarterial chemoembolization; HR, hazard ratio; ITT, intent-to-treat; OS, overall survival; PFS, progression-free survival; PP, per-protocol.

In the ITT population, patients in the DEB-TACE group had a median PFS of 6.0 months, compared with 4.0 months for those in the cTACE group [hazard ratio (HR) 0.62; 95% CI, 0.42–0.92; *P*=0.018; Fig. 3A]. Furthermore, patients in the DEB-TACE group exhibited a significantly longer median OS of 12.0 months (95% CI, 9.0–15.0), compared with 7.0 months (95% CI, 6.0–10.0) for those in the cTACE group (HR 0.68; 95% CI, 0.48–0.96; *P*=0.027; Fig. 3B). In the PP population, patients in the DEB-TACE group exhibited a median PFS of 6.0 months, compared with 4.0 months for those in the cTACE group (HR 0.63; 95% CI, 0.42–0.95; *P*=0.027; Fig. 3C). Additionally, patients in the DEB-TACE group exhibited a significantly longer median OS of 12.0 months (95% CI, 9.0–16.0), compared with 8.0 months (95% CI, 7.0–11.0) for those in the cTACE group (HR 0.69; 95% CI, 0.48–0.98; *P*=0.039; Fig. 3D).

Univariate analysis showed that for the PP population, the treatment group (*P*=0.027), ALBI score (*P*=0.022), LTB (*P*=0.004), distant metastasis (*P*=0.036) and additional ICIs (*P*=0.018) or TKIs (*P*=0.004) were significant prognostic factors for PFS, while the treatment group (*P*=0.039), ALBI score (*P*=0.005), LTB (*P*=0.002), distant metastasis (*P*=0.039), PVTT group (*P*< 0.001) and combined surgery (*P*< 0.001) were significant prognostic factors for OS. The PVTT group and combined surgery also strongly influenced OS (all *P*<0.001). Moreover, multivariate analysis revealed that the treatment group, ALBI score, distant metastasis and additional TKIs were independent prognostic factors for PFS, while the treatment group, PVTT group, and combined surgery were three significant independent prognostic factors for OS (Tables 5 and 6).

**Table 5:** Results of univariate and multivariate cox regression analysis for PFS in PP population.

		Univariate analysis		Multivariate analysis	
Variants		Hazard ratio (95% CI)	*P*	Hazard ratio (95% CI)	*P*
Treatment group	DEB-TACE	0.63 (0.42–0.95)	0.027	0.6 (0.4–0.92)	0.018
Age group	≥60	0.9 (0.58–1.4)	0.635		
Sex	Male	0.94 (0.48–1.81)	0.844		
ECOG	1	1.47 (0.96–2.27)	0.077		
Child-Pugh stage	B	1.35 (0.78–2.32)	0.28		
ALBI score	Grade2	1.61 (1.07–2.43)	0.022	1.68 (1.08–2.6)	0.020
Tumor number	Multiple	1.46 (0.96–2.21)	0.075		
Tumor size group	≤10	0.6 (0.32–1.13)	0.112		
	>10	0.92 (0.5–1.7)	0.787		
LTB	>50%	1.81 (1.21–2.7)	0.004	1.45 (0.95–2.22)	0.088
PVTT group	III/IV	1.27 (0.83–1.92)	0.267		
PVTT supply	Rich	0.82 (0.55–1.22)	0.328		
Distance metastasis	Yes	1.56 (1.03–2.36)	0.036	1.76 (1.12–2.76)	0.014
Hepatic vein invasion	Yes	1.29 (0.86–1.95)	0.22		
AFP group	<400	0.89 (0.5–1.58)	0.697		
	≥400	0.88 (0.53–1.44)	0.601		
Plus ICIs	Yes	0.6 (0.4–0.92)	0.018	0.68 (0.42–1.1)	0.115
Plus TKIs	Yes	0.55 (0.36–0.82)	0.004	0.58 (0.37–0.91)	0.018
Plus TCM	Yes	0.72 (0.48–1.1)	0.134		

AFP, alpha-fetoprotein; ALBI grade, Albumin-Bilirubin grade; DEB-TACE, drug-eluting bead transarterial chemoembolization; ECOG, Eastern Cooperative Oncology Group; ICIs, immune checkpoint inhibitors; LTB, liver tumor burden; PFS, progression-free survival; PP, per-protocol; PVTT, portal vein tumor thrombosis; TCM, Traditional Chinese Medicine; TKIs, tyrosine kinase inhibitors.

**Table 6:** Results of univariate and multivariate cox regression analysis for OS in PP population.

		Univariate analysis		Multivariate analysis	
Variants		Hazard ratio (95% CI)	*P*	Hazard ratio (95% CI)	*P*
Treatment group	DEB-TACE	0.69 (0.48–0.98)	0.039	0.69 (0.48–1)	0.047
Age group	≥60	0.84 (0.57–1.23)	0.367		
Sex	Male	1.03 (0.55–1.92)	0.921		
ECOG	1	1.39 (0.96–2.01)	0.085		
Child-Pugh stage	B	1.47 (0.91–2.38)	0.117		
ALBI score	Grade2	1.68 (1.17–2.42)	0.005	1.43 (0.97–2.1)	0.069
Tumor number	Multiple	1.42 (0.99–2.05)	0.059		
Tumor size group	≤10	0.96 (0.51–1.78)	0.894		
	>10	1.41 (0.75–2.65)	0.289		
LTB	>50%	1.78 (1.24–2.55)	0.002	1.11 (0.74–1.65)	0.625
PVTT group	III/IV	2.13 (1.46–3.09)	<0.001	1.79 (1.22–2.61)	0.003
PVTT supply	Rich	1.21 (0.85–1.73)	0.293		
Distance metastasis	Yes	1.49 (1.02–2.18)	0.039	1.32 (0.89–1.97)	0.168
Hepatic vein invasion	Yes	1.31 (0.91–1.88)	0.149		
AFP group	<400	1.32 (0.78–2.23)	0.309		
	≥400	1.48 (0.93–2.35)	0.096		
Plus ICIs	Yes	0.78 (0.54–1.13)	0.191		
Plus TKIs	Yes	1.19 (0.83–1.7)	0.353		
Plus TCM	Yes	0.71 (0.49–1.04)	0.076		
Combined SBRT	Yes	1.07 (0.67–1.71)	0.769		
Combined surgery	Yes	0.14 (0.06–0.3)	<0.001	0.18 (0.08–0.39)	<0.001

AFP, alpha-fetoprotein; ALBI grade, Albumin-Bilirubin grade; DEB-TACE, drug-eluting bead transarterial chemoembolization; ECOG, Eastern Cooperative Oncology Group; ICIs, immune checkpoint inhibitors; LTB, liver tumor burden; OS, overall survival; PP, per-protocol; PVTT, portal vein tumor thrombosis; TCM, Traditional Chinese Medicine; TKIs, tyrosine kinase inhibitors.

### Safety

The incidence of any grade of AEs was similar between the DEB-TACE and cTACE groups. The most frequent AE observed was post-embolization syndrome (PES), which included nausea and vomiting in 71 (87%) patients in the DEB-TACE group and 72 (89%) in the cTACE group, abdominal pain in 70 (85%) patients in the DEB-TACE group and 74 (91%) in the cTACE group, and fever in 52 (63%) in the DEB-TACE group and 42 (52%) in the cTACE group. The frequencies of grade 3–4 elevated ALT were significantly higher in the cTACE group [28 (34.6%)] than in the DEB-TACE group [17 (20.7%)] (*P* =0.048) (Table 7). Two patients died within 30 days of the procedure. One patient in the DEB-TACE group died from liver abscess, while another in the cTACE group died of acute liver failure with grade 4 hyperbilirubinemia.

**Table 7:** All-grade treatment-emergent adverse events within 1 week after first TACE in ITT population.

Adverse event	Overall, *N*=163, *N* (%)^a^	cTACE, *N*=81, *N* (%)^a^	DEB-TACE, *N*=82, *N* (%)^a^	*P* ^b^
Nausea/vomiting	143 (88)	72 (89)	71 (87)	0.65
Abdominal pain	144 (88)	74 (91)	70 (85)	0.23
Fever	94 (58)	42 (52)	52 (63)	0.14
Leukopenia				1.000
grade 1–2	162 (99.4)	81 (100)	81 (99.4)	
grade 3–4	1 (0.6)	0	1 (1.2)	
Neutropenia				1.000
grade 1–2	162 (99.4)	81 (100)	81 (98.8)	
grade 3–4	1 (0.6)	0	1 (1.2)	
Anemia				1.000
grade 1–2	161 (98.8)	80 (98.8)	81 (98.8)	
grade 3–4	2 (1.2)	1 (1.2)	1 (1.2)	
Thrombocytopenia				0.534
grade 1–2	152 (93.3)	77 (95.1)	75 (91.5)	
grade 3–4	11 (6.7)	4 (4.9)	7 (8.5)	
Hypoalbuminemia				1.000
grade 1–2	163 (91)	81 (100)	82 (100)	
grade 3–4	0	0	0	
Elevated ALT				**0.048**
grade 1–2	118 (72.4)	53 (65.4)	65 (79.3)	
grade 3–4	45 (27.6)	28 (34.6)	17 (20.7)	
Elevated AST				0.071
grade 1–2	92 (56.4)	40 (49.4)	52 (63.4)	
grade 3–4	71 (43.6)	41 (50.6)	30 (36.6)	
Hyperbilirubinemia				0.131
grade 1–2	152 (93.3)	73 (90.1)	79 (96.3)	
grade 3–4	11 (6.7)	8 (9.9)	3 (3.7)	
Thirty-day mortality	2 (1.2)	1 (1.2)	1 (1.2)	1.000

ALT, alanine aminotransferase; AST, aspartate aminotransferase; cTACE, conventional transarterial chemoembolization; DEB-TACE, drug-eluting bead TACE; IQR, interquartile range; ITT, intent-to-treat.

a Median (IQR) or frequency (%).

b Pearson’s χ^2^ test; Fisher’s exact test.

## Discussion

Currently, it is widely acknowledged that TACE represents a relatively safe and effective therapeutic approach for HCC patients with PVTT, as described in the LAUNCH trial^23^. The LAUNCH trial findings suggest that TACE exhibits promising efficacy as a therapeutic approach for HCC patients with PVTT. In the Lenvatinib plus TACE group, a significant proportion (71.8%) of patients had PVTT, and the clinical outcomes of this combination treatment were superior to Lenvatinib monotherapy^23^. However, whether DEB-TACE is superior to conventional TACE (cTACE) remains unclear. In our randomized controlled trial, we evaluated the efficacy and safety in patients with HCC and PVTT who underwent DEB-TACE therapy compared to those who received cTACE. We found that patients in the DEB-TACE group had a higher overall ORR (66.5% vs. 46.6%; *P*=0.015) but a similar DCR (92.2% vs. 86%, *P*=0.2) compared to those in the cTACE group. We used PFS as the primary endpoint instead of OS in this trial for it was less vulnerable to subsequent treatments after progression^24^ and our result showed a statistically significant improvement in the PFS for DEB-TACE versus cTACE (6.0 vs. 4.0 months, HR 0.69, 95% CI, 0.48–0.98, *P*=0.039). Moreover, we also found that patients in the DEB-TACE group had significantly longer OS than those in the cTACE group (12.0 vs. 8.0 months, HR 0.63, 95% CI, 0.42–0.95, *P*=0.027) with comparable safety profiles. Therefore, DEB-TACE may be a promising therapeutic approach for HCC patients with PVTT, providing a higher ORR and significant survival benefit.

Systemic therapies, such as TKIs and ICIs, are commonly recommended as the standard treatment for advanced HCC. However, the survival advantages for patients with PVTT are somewhat limited. In two randomized phase III studies, Sorafenib showed a statistically significant survival benefit when compared to placebo in advanced HCC (SHARP study^25^ and Asia-Pacific study^26^). Nevertheless, it’s worth noting that only 36% of patients in the sorafenib group exhibited macrovascular invasion in those studies, and the PFS ranged from only 2.8–5.5 months. Despite recent breakthroughs in systemic treatments for advanced HCC, particularly the combination of TKIs and ICIs, the survival benefits for patients with PVTT remain unsatisfactory. The sub-analysis of the IMbrave 150 trial demonstrated that atezolizumab plus bevacizumab resulted in median PFS and OS of 6.7 months and 14.2 months for HCC patients with macrovascular invasion, respectively^27^. The results of IMbrave 150 are promising; and were superior to the 6.0 months and 12.0 months achieved with DEB-TACE in our study. However, the ORR was only 27% and the incidence of AEs was high at 76%, including 56.5% severe events. Therefore, the survival benefits of the IMbrave 150 trial may be attributed to effective subsequent treatments^27^. Updated data from the HIMALAYA study presented at the American Society of Clinical Oncology 2022 annual meeting showed a PFS of only 3.8 months and a median OS of 16.4 months for durvalumab plus tremelimumab treating unresectable HCC^28^. The longer median OS in that study could potentially be attributed to the fact that only a minority (26.2%) of patients had macrovascular invasions. A recent multicenter randomized controlled trial showed that sorafenib plus cTACE for patients with HCC and main trunk PVTT had a median PFS of 4.2 months and a median OS of 6.3 months^29^, which were inferior to the treatment of irradiation stent with 125 I plus cTACE (6.6 months and 9.9 months, respectively).These results illustrate that the efficacy of local treatments can be comparable to that of systemic treatment in treating HCC with PVTT, as shown by our results.

In our study, we investigated various predictors of PFS and OS. We found that several factors, including treatment group, ALBI score, LTB, presence of distant metastasis, and additional TKIs, were significant prognostic factors associated with PFS. Like many other cancers, the presence of distant metastasis and high LTB is the main contributor to poor prognosis^30^.The ALBI score is an objective measure that can detect subtle changes in liver dysfunction more effectively than the Child-Pugh or MELD scores^31^. Hence, the ALBI score is considered a powerful tool for improving treatment options and has been extensively reported and summarized for its prognostic value in HCC treatments^31,32^. ALBI score also predicts survival, toxicity, and post-procedural liver failure in patients treated with TACE. A recently research found it to be superior to Child-Pugh classification in distinguishing overall survival among HCC patients undergoing DEB-TACE^33^. In this study, we also observed that patients with ALBI grade 1 had better PFS outcomes compared to those with ALBI grade 2 (*P* value<0.05). Two recent large-scale multicenter randomized controlled trials, known as the TACTICS trial^34^ and the LAUNCH trial^23^, have demonstrated that the addition of TKIs to TACE can enhance the therapeutic efficacy of TACE and extend PFS significantly. The treatment group was an independent prognostic factor significantly associated with PFS and OS, indicating that DEB-TACE offers superior survival benefits compared to cTACE. Additionally, in our study, PVTT group and combined surgery were strong predictors of OS. While previous studies have shown that the extent of PVTT is less critical than its presence^2^, our results demonstrate that patients with type I/II PVTT had significantly better survival compared to those with type III/IV PVTT.

The overall incidence of AEs was comparable between the two groups, and severe AEs were infrequent. PES was the most frequently observed AEs among patients in this study, consistent with our previous findings^35^. Notably, the frequencies of grade 3–4 elevated ALT and aspartate aminotransferase were slightly higher in the cTACE group compared to the DEB-TACE group (*P* =0.048 and 0.071, respectively). These results indicated that cTACE may have a slightly greater impact on liver function compared to DEB-TACE, which may explain why one patient in the cTACE group experienced acute liver failure and early death. It should be noted that DEB-TACE has been associated with a higher incidence rate of bile duct injury and tumor necrosis compared to cTACE^36^, and in the current study, one death was attributed to liver abscess caused by DEB-TACE. However, DEB-TACE has been shown to enhance immune cell infiltration in tumor tissues, which enhances the efficacy of systemic therapies like immunotherapies^37^, and leads to better PFS and tumor response rates^38^. Therefore, we recommend prophylactic antibiotics for high-risk patients before undergoing DEB-TACE^18^.

Several limitations to this study should be acknowledged. Firstly, the original study design only considered the grade of portal vein invasion as a randomized stratification factor, neglecting other factors such as tumor size, LTB, and hepatic vein invasion. Consequently, the cTACE group exhibited larger tumors, higher LTB, and more instances of hepatic vein invasion compared to the DEB-TACE group, although these factors were statistically balanced. Consequently, future clinical and basic research should incorporate these factors to design and analyze research data. Secondly, as systemic therapies were available to patients, including TKIs, ICIs, and TCM, these treatments may have influenced the outcomes, despite no statistical difference between the two groups. Third, patients with distant metastasis or hepatic vein invasion were not excluded, which may limit the improvement of TACE efficacy and survival benefits. Finally, patients with obvious arteriovenous fistulas were not included in the study, and further research is needed to evaluate the value of DEB-TACE in these patients. Despite these limitations, this study provides valuable insights into the potential efficacy of DEB-TACE as a treatment option for HCC patients with PVTT.

## Conclusion

This study demonstrated that DEB-TACE is superior to cTACE in treating HCC patients with PVTT due to the improved PFS and OS with an acceptable safety profile and may thus become a promising treatment strategy for HCC patients with PVTT.

## Ethical approval

The study protocol was conducted in accordance with the principles of the World Medical Association Declaration of Helsinki and was approved by the ethics committee of the First Affiliated Hospital, Zhejiang University School of Medicine (Approval Numbers: 2018-804). The written informed consent was obtained from all patients.

## Consent

Written informed consent was obtained from the patient for publication of this case report and accompanying images. A copy of the written consent is available for review by the Editor-in-Chief of this journal on request.

## Source of funding

This work was supported by National Natural Science Foundation of China (Grant No. 82102160), and Research Fund for Interventional Oncology of China Health Promotion Foundation (No.XM_2018_011_0006_01).

## Author contribution

T.-Y.Z.: conceptualization, data curation, investigation, methodology, software, validation, visualization, funding acquisition, writing—original draft, and writing—review and editing; G.-F.T.: conceptualization, data curation, investigation, methodology, resources, validation, and visualization; writing—original draft, and writing—review and editing; G.-H.Z.: conceptualization, data curation, investigation, methodology, validation, visualization, writing—original draft, and writing—review and editing; Y.-L.Z.: conceptualization, investigation, resources, validation, and visualization; S.-Q.C.: conceptualization, investigation, methodology, resources, validation, and visualization; Y.-L.Z., T.-Y.Z., B.-Q.W., S.-Q.C. and H.-L.W.: conceptualization and methodology; L.J.: investigation, methodology, formal analysis, software and funding acquisition; F.C.: conceptualization, formal analysis, investigation, methodology, resources, software, validation, visualization, and writing—review and editing.

## Conflicts of interest disclosure

All authors declare no potential conflicts of interest.

## Research registration unique identifying number (UIN)

1. Name of the registry: Clinical application research of drug-eluting beads transarterial chemoembolization with CalliSpheres beads for advanced hepatocellular carcinoma with portal vein tumor thrombus.
2. Unique identifying number or registration ID: ChiCTR1800018035.
3. Hyperlink to your specific registration (must be publicly accessible and will be checked): https://www.chictr.org.cn/showproj.html?proj=30495.

## Guarantor

Tan-Yang Zhou and Feng Chen.

## Data availability statement

All data generated or analyzed during this study are included in this article. Further inquiries can be directed to the corresponding authors.

## Provenance and peer review

Not commissioned, externally peer-reviewed.
